# A Kalman-Filter Based Approach to Identification of Time-Varying Gene Regulatory Networks

**DOI:** 10.1371/journal.pone.0074571

**Published:** 2013-10-07

**Authors:** Jie Xiong, Tong Zhou

**Affiliations:** 1 Department of Automation, Tsinghua University, Beijing, China; 2 Department of Automation and Tsinghua National Laboratory for Information Science and Technology(TNList), Tsinghua University, Beijing, China; Memorial Sloan Kettering Cancer Center, United States of America

## Abstract

**Motivation:**

Conventional identification methods for gene regulatory networks (GRNs) have overwhelmingly adopted static topology models, which remains unchanged over time to represent the underlying molecular interactions of a biological system. However, GRNs are dynamic in response to physiological and environmental changes. Although there is a rich literature in modeling static or temporally invariant networks, how to systematically recover these temporally changing networks remains a major and significant pressing challenge. The purpose of this study is to suggest a two-step strategy that recovers time-varying GRNs.

**Results:**

It is suggested in this paper to utilize a switching auto-regressive model to describe the dynamics of time-varying GRNs, and a two-step strategy is proposed to recover the structure of time-varying GRNs. In the first step, the change points are detected by a Kalman-filter based method. The observed time series are divided into several segments using these detection results; and each time series segment belonging to two successive demarcating change points is associated with an individual static regulatory network. In the second step, conditional network structure identification methods are used to reconstruct the topology for each time interval. This two-step strategy efficiently decouples the change point detection problem and the topology inference problem. Simulation results show that the proposed strategy can detect the change points precisely and recover each individual topology structure effectively. Moreover, computation results with the developmental data of *Drosophila Melanogaster* show that the proposed change point detection procedure is also able to work effectively in real world applications and the change point estimation accuracy exceeds other existing approaches, which means the suggested strategy may also be helpful in solving actual GRN reconstruction problem.

## Introduction

Identifying causal relationships of a gene regulatory network (GRN) is one of the fundamental problems in understanding cell behaviors. For most conventional identification methods, it is generally assumed the topological structure is constant over time. Based on this assumption, various models and methods have been proposed, such as Boolean networks [1], Bayesian networks [2], regression and correlation analyses based methods [3], ordinary differential equation (ODE) based methods [4], etc.

Recent research results, however, show that GRNs are dynamic in response to physiological and environmental changes. For instance, an example of such time-varying regulatory network can be provided by the development of the fruitfly *Drosophila Melanogaster*, which is segmented into different life stages: embryogenesis, larva, pupa and adult [5]. Moreover, some studies have also confirmed that the active regulatory paths in a gene expression network of *Saccharomyces cerevisiae* exhibit dramatic topological changes and hub transience during a temporal cellular process and in response to diverse stimuli [6]. Although there is a rich literature in modeling static or temporally invariant networks, how to systematically recover these temporally changing networks remains a major and significant pressing challenge.

To identify time-varying GRNs, some special methods have been proposed recently. A machine learning method called TESLA is presented in [7], which builds on a temporally smoothed 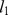-regularized logistic regression formalism that can be cast as a standard convex-optimization problem and solved by using generic solvers scalable to large networks. However, the estimated topology by this method is undirected and suboptimal. In addition, there exist some methods that follow the Bayesian paradigm [8]–[11]. While these approaches also have their limitations. The method suggested in [8] assumes a fixed network structure and only allows the interaction parameters to vary with time, which is too rigid and idealistic in practice. The method proposed in [9] requires a discretization of the data, which incurs an ineluctable information loss. And, the limitation in [10], [11] is that these methods need prior distributions on the network structure.

The purpose of this study is to suggest a two-step strategy that recovers time-varying GRNs. In this paper, the model for time-varying GRNs is adopted as the switching auto regressive model. Consequently, in the first step, on the basis of a relation between the Kalman filter and recursive least squares (RLS) estimation, it is shown that a stochastic process can be constructed which is white if and only if the time series expression data are generated by the same sub-regulatory network. Based on this observation, a procedure is developed to detect change points of a time-varying GRN. The observed time series are divided into several segments using these detection results; and each time series segment belonging to two successive demarcating change points is associated with an individual static regulatory network. And then, in the second step, conditional network structure identification methods are used to reconstruct the topology for each time interval. In summary, in the suggested time-varying GRN identification strategy, the problem of identifying a time-varying regulatory network is transformed into that of identifying multiple single static regulatory networks. To solve the latter is much easier than to solve the former. For the performance evaluation, we use both time series data generated by a synthetic time-varying GRN and time series data provided by the DREAM3 challenge, and simulation results confirm that the proposed strategy can detect the change points precisely and recover each individual topological structure effectively. For a real data application, our proposed strategy is applied to time series data of *Drosophila Melanogaster* during a complete time-course of development, and computation results show that the proposed change point detection procedure has ability to work effectively in real world applications and the change point positioning accuracy exceeds other existing approaches, which means the suggested strategy may also be helpful in solving actual GRN reconstruction problem.

The rest of this paper is organized as follows. At first, the problem discussed in this paper and some mathematic preliminary results are given, then the change points estimation algorithm is illustrated and the two-step strategy is derived. Afterwards, the proposed estimation strategy is assessed using both *In Silico* data and the developmental data of *Drosophila Melanogaster*. Variations of estimation performances with respect to parameters of the suggested method will also be reported. Besides, some concluding remarks are given about the characteristics of the suggested method, as well as some future works worthy of further efforts. Finally, an appendix is included in Text S1 to give proof of some technical results.

The following notations are adopted in this paper. 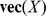 denotes the operation of stacking the columns of matrix 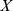 from left to right, and 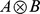 the Kronecker product of matrices 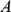 and 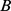. 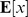 and 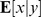 stand respectively for the expected value of a random variable 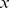 and the conditional expected value of a random variable 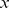 given an observation of the random variable 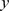. 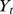 is defined as 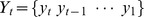, while 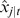 represents an estimate about 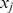 based on some observed data from 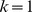 to 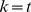. Both 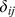 and 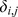 are used to denote the Kronecker delta function. To avoid an awkward presentation, no difference is made in this paper between a random variable and its realizations.

## Methods

### Problem Statement and Preliminary Results

Generally speaking, a basic model for a time-varying GRN consisting of 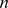 genes can be expressed as [12]

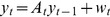
(1)Here, 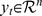 is the time series experiment data of gene expression, and 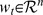 is the Guassian white noise as 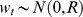. The system matrix 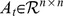 captures the causal relationships of genes, that is, if 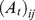 is positioned significantly far from zero, the 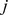-th gene captures a large effect on the 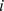-th gene from time point 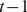 to 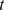. On the other hand, it is generally assumed that the regulatory mechanism among genes is unlikely to change drastically over small time intervals [7], [13], which presumes that the coefficient matrix 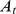 should vary smoothly with time. Based on this assumption, Equation (1) can be expressed as the switching auto regressive (SAR) model as follows:
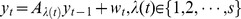
(2)By means of the model described by Equation (2), some concepts are given. The so-called “change point” means that the time instant at which its discrete state 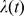 changes value, and the time series segment belonging to two successive demarcating change points is associated with an individual static regulatory network which the causal relationship can be captured by 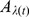.

It has been pointed out that over the course of a cellular process, such as a cell cycle or an immune response, there may exist multiple underlying themes that determine the functionalities of each molecule and their relationships to each other, and such themes are dynamic and stochastic [7]. As a result, GRNs are dynamic in response to physiological and environmental changes.

In general, normal biological tissues will undergo morphologic changes when they are inflicted by some external stimuli, such as ionizing radiations. In fact, many literatures have studied the radiation tolerance [14]–[16], especially, the “Time-Dose” relationships [17]; that is to say, the time span that the normal biological networks remain unchanged when they are eroded by certain amount of ionization radiation is unknown. In other words, the change points are not known *a priori* in this type experiment. And by extension, the change points are not always known *a priori* in general. Therefore, it is assumed that the change point is unknown and its value is needed to estimate in some literatures on the identification of time-varying GRNs [10], [11]. We also hold this assumption in the paper.

Based on the discussion above, the time-varying GRN identification problem discussed in this paper is as follows.

#### Problem

Given a series of gene expression vectors 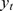 generated by model (2), 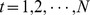, estimate all the change points, the number of sub-networks 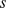, and the model parameters 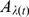, 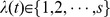.

It is well known that the computation procedure of recursive least squares (RLS) parametric estimations for AR models possesses the same form as that of Kalman filtering [18], [19]. Using these similarities, some system identification problems can be easily transformed into a state estimation one, and vice versa. To investigate the above change point estimation problem, some relations are introduced here between RLS estimations of an AR system and Kalman filtering.

Consider the following linear time invariant (LTI) AR system
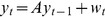
(3)in which 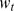 is a sequence of independent random vectors with zero mean and covariance matrix 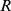. Rewrite Equation (3) into a state-space form as follows,
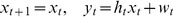
(4)Here, 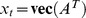, 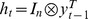. Assume that 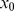 is an *a priori* unbiased estimate about 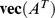 and its covariance matrix is 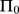. Moreover, assume that this estimate is independent of 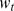 with 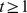. Through adopting the general results of [18], [19] on relations between RLS parametric identification and state estimation to the above LTI AR system, the following results can be straightforwardly obtained.

#### Lemma 1

Set 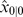 and 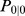 respectively as 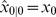 and 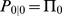. Based on gene expression time series data 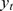 generated by model (3), 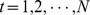, the RLS estimate for its model parameters 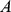, denote it by 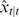, can be recursively computed as follows,
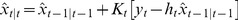
(5)

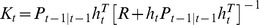
(6)

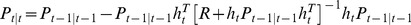
(7)Moreover, if both 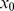 and 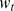 are normally distributed, then, 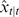 is also normally distributed, and 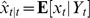.

### Change point Detection Procedure

In the time-varying GRN identification, a common situation is that available knowledge about the actual network topology is nothing but its gene expression data. In order to develop the change points detection procedure, it appears appropriate to investigate at first whether or not there exist some detectable stochastic differences between gene expression data generated by the same sub-network and those generated by more than one sub-network. If the answer is positive, then a change of these stochastic properties reflects a switch between two sub-networks. In other words, a statistic can be constructed for estimating change points of a time-varying GRN. Based on these considerations, stochastic properties of an innovation process of the network described by Equation (2) are investigated with respect to the recursive estimation procedure given by Equations (5)–(7), in case that gene expression data are generated respectively by a single sub-network and multiple sub-networks.

#### Theorem 1

Suppose that gene expression time series data 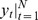 are generated by the time-varying GRN described by Equation (2). On the basis of the recursive estimation procedure of Equations (5)–(7), define an innovation process 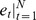 as follows,
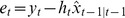
(8)Then, 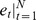 is an independent random sequence if and only if the network described by Equation (2) collapses to a static network.

A proof of the above theorem is given in Text S1. Furthermore, from the above discussions, a direct result of Equation (2) is that 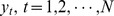, is also normally distributed. On the other hand, from properties of Kalman filtering, it can be declared that 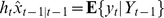. Therefore, 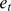 defined in Equation (8) is a normally distributed random vector. On the basis of Theorem 1 and its extensions, the following results can be obtained through straightforward algebraic manipulations. These results are very helpful in detecting the change point.

#### Corollary 1

Assume that for arbitrary 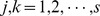 and 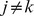, there exists at least one scenario that 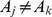. For gene expression time series data 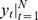 generated by model (2), define 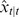 recursively using the procedure of Equations (5)–(7). Moreover, define a time series 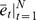 as follows
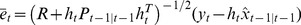
(9)Then, 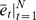 is a sequence of independently distributed random variables with zero mean and unit covariance matrix, if and only if there is no sub-network switch during the time period 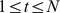.

Corollary 1 makes it clear that change point estimation for a time-varying GRN described by Equation (2) can be transformed to independence validation of a Gaussian random sequence. The latter can be checked by chi-square test. Based on the definition of the 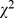-distribution, we have the following result.

#### Corollary 2

Based on the conditions of Corollary 1, define 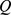 as
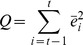
(10)Then, 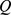 obeys the 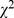-distribution with degrees of freedom 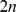, if and only if there is no sub-network switch during the time period 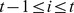.

The results of Corollary 2 are helpful in detecting the change point. As a matter of fact, in actual applications, if 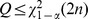, then, the hypothesis that the collected gene expression data are generated by the same sub-network can not be rejected with a confidence level 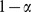. Based on the results of Corollary 1 and Corollary 2, a procedure can be developed for detecting the change point. Details of this procedure are given in Table 1.

**Table 1:** Change point Detection Procedure.

S1:	Initialization: Select a positive number 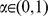 . Set 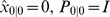 .
S2:	Calculate recursively 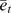 using Equation (9) and the procedure of Equations (5)–(7).
	If the number of the computed 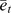 is greater than 2, compute the statistic *Q*.
S3:	If 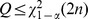 , the current time instant isn't a change point. Let 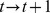 and return
	to S2. Otherwise, record the current time instant as a change point, assign it to be the
	initial time for detecting the next change point, and return to S1.
S4:	Stop the procedure in case that every gene expression data has been utilized.

An attractive property of the change point detection procedure is that its computational complexity does not depend on the number of change points. Moreover, it is also worthwhile to point out that in this detection procedure, neither prior distribution on the number of change points nor knowledge about the change time instant is required, i.e., our change points detection procedure do not require the structure prior distribution of a GRN, which is the major difference from the method proposed in [10], [11].

### Two-Step Strategy

In the above subsection, the change point estimates have been obtained, which are denoted by 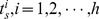. Base on these change point estimates, the observed gene expression time series data are divided into 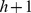 segments, which are 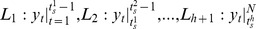; and each time series segment belonging to two successive demarcating change points is supposed to associate with an individual static GRN. Consequently, for each time interval, the causal relationships inference problem can resort to conditional network structure identification methods. The suggested two-step strategy inference method for time-varying GRNs is summarized as follows.

1. Estimate change points using the change point detection procedure in Table 1.
2. For each time series segment, infer the causal relationships by conditional network structure identification methods, such as IOTA [20], and LASSO [21], etc.

In summary, the suggested two-step strategy can decouple the change point detection problem and the topology inference problem, that is, the problem of identifying a time-varying regulatory network is transformed into that of identifying multiple single static regulatory networks. To solve the latter is much easier than to solve the former.

#### Remark

It should be noted that the number of the biological experimental time series data is very limited. If there exist some *a priori* information about the network topology, it is also desirable to jointly learn the static networks across time segments. A feasible method is as follows.

Suppose that the time series 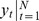 is cut into two segments 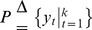, and 
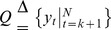
 by the suggested change point detection procedure. If gene 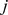 regulate gene 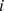 throughout this time period based on other *a priori* knowledge, then we can set the initial value 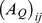 as 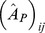 that is obtained from the data segment 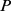, when learn the network topology based on the data segment 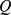. Therefore, jointly learning the static networks across time segments can be done by this way.

## Results and Discussion

### Simulation Study

In order to evaluate the properties of the suggested two-step strategy, gene expression time series data are generated by an academic dynamic network. This simulated dynamic network include two sub-networks denoted by 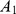 and 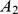. The simulation time span is 60, and at time instant 31, the active sub-network is changed from 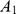 to 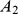. The simulated dynamic network include 10 genes; and the nonzero elements for 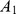 are 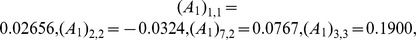 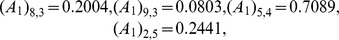 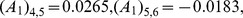 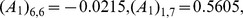 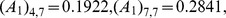 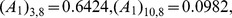 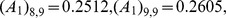 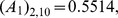 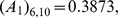 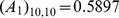 respectively; while the nonzero elements for 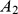 are 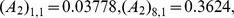 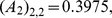 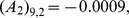 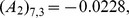 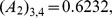 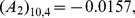 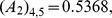 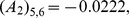 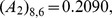 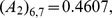 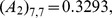 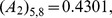 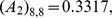 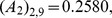 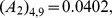 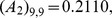 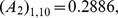 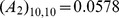, respectively. The noise 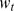 is a sequence of independent Gaussian random variable with mean 2 and variance 0.5.

In the second step, we apply a recent identification method, named the inner composition alignment (IOTA) [20]. In IOTA, a measurement 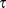 is defined to characterize the causality of two time series. For the given short time series 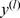 and 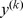, sort 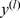 with the order 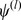, such that 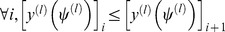, and reorder the time series 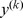 with respect to 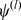 as 
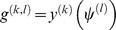
. Define 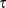 as follows,
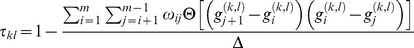
Here, 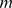 is the length of the time series, 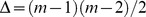 is a normalization constant which corresponds to the maximum number of crossings, 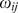 denotes a weight, and 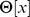 is the Heaviside step function,
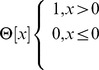
In the last ranking list of 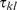 , if the magnitude is larger, the corresponding transcription regulation will be established in a larger probability from gene *l* to gene *k*.

In systems biology, predictions are compared with the actual network structure using the following two different metrics in topology prediction accuracy evaluations.

- AUPR: The area under the precision-recall curve;
- AUROC: The area under the receiver operating characteristic curve.

Choose *α* as 0.05, independently simulate this dynamic network 500 times, and the results are summarized in Table 2.

**Table 2:** Performances with simulation data.

	changetime estimate	AUROC1	AUPR1	AUROC2	AUPR2
mean	31.0900	0.6208	0.4043	0.6454	0.3414
standard deviation	0.2865	0.0410	0.0588	0.0417	0.0476

The change point estimated mean in Table 2 are very close to the actual change time instant, and the variance of these estimates is very small, which shows our change point detection procedure is effectively. And, the estimate variances of AUROC and AUPR are quite small. It is concluded that the main difficulty of the switched autoregressive exogenous model identification is that the identification problem includes a classification problem in which each data point must be associated to the most suitable sub-model; and, the more precise the data classification is, the better the identification results are [22]. This argument is also suitable for the scenario of identifying a time-varying GRN. Due to the high accuracy of the change point estimates, the estimate variances of AUROC and AUPR are quite small. Therefore, these simulation results show that our two-step strategy is appropriate for reconstructing time-varying GRNs.

The above simulation is an academic case, in which the experimental environment is very close to the fundamental assumption in the section of Methods. In the rest of this subsection, we will give another simulation, in which gene expression data are from the DREAM3 *in silico* size10 challenge [23], [24]. Although gene expression data in the DREAM challenges are emulational, the simulation model in the DREAM challenges is nonlinear and the noise is not exactly Gaussian. Therefore, the source networks are closer to the real situation. Due to the static nature of the network in the DREAM challenges, we concatenate gene expression data generated by 5 different networks to simulate a time-varying GRN. Applying our strategy to these data, the simulation results are shown in Table 3.

**Table 3:** Performances with DREAM3 *in silico* size10 challenge.

segment number	starting point (actual/estimate)	ending point (actual/estimate)	AUROC	AUPR
1	1/1	21/21	0.7344	0.2007
2	22/22	42/42	0.6933	0.2893
3	43/43	63/63	0.6011	0.1354
4	64/64	84/84	0.6789	0.3522
5	85/85	105/105	0.7681	0.4408

From Table 3, we know the actual change time instants are 22, 43, 64, 85, respectively, and our change point detection procedure estimate these change points accurately. That is, gene expression time series are successfully divided into five segments, and the problem of identifying a time-varying regulatory model is effectively transformed into that of identifying five single regulatory models. Consequently, conditional identification methods for the static GRN are used to reconstruct the topology for each segment, which means that the proposed two-step strategy can ease the difficulty level in recovering a time-varying GRN.

### Real Data Application

The gene expression data of *Drosophila Melanogaster* have been well-studied in different aspects [5], [10], [11], [25]. Here, we apply our two-step strategy to the developmental data provide by [5]. In this study, the researchers have reported gene expression data for nearly one third of all *Drosophila Melanogaster* genes during a complete time-course of development. And, cDNA microarrays were used to analyze the RNA expression levels during 66 sequential time periods, including the embryonic period (30 samples), the larval period (10 samples), the pupal period (18 samples) and the first 30 days of adulthood (8 samples). In addition, a major morphological change relates to a modification of transcriptional regulations during the first 0 to 6.5 hours of embryonic development, which consists of 12 samples. Therefore, the actual change instants ought to be 13, 31, 41, and 59. Here, we use a sub-dateset of this developmental data, containing the following 11 genes: ‘actn’,‘eve’,‘gfl’,‘mhc’,‘mlc1’,‘msp300’,‘myo61f’,‘prm’,‘sls’,‘twi’, and ‘up’.

To apply our change point detection procedure, we first estimate noise covariance matrix 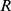. Reformulize the basic model (1) as
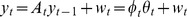
Here, 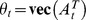, 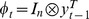. Based on this formulation, we utilize the weighted recursive least square algorithm to estimate 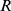 [18], [19]. Concretely, given the initial condition 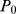 and 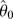, the recursive expression equations to calculate 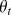 are given as follows.
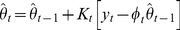
(11)

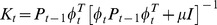
(12)

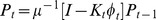
(13)On the basis of the weighted recursive least square procedure of Equations (11)–(13), define a residual sequence 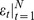 as follows,
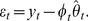
(14)Then, the noise covariance matrix 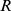 can be estimated as
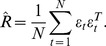
(15)The typical initial value can be selected as 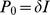, where 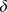 is a large number, 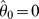, and the weighting coefficient 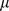 is typically chosen as 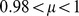 [18], [19].

By setting 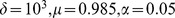, the change point estimates by the suggested change point detection procedure are 13, 31, 46, 59. Although the third change point estimate is a little away from the actual value, the other changetime estimates are equal to the actual change instants. On the other hand, using the same dataset, a report that time intervals {18 to 19}, {31 to 33}, {41 to 43} and {59 to 61} contain more than 40% of the change points can be found in [10]; and in [11], the authors have given only the last three change points. These results show that the proposed change point detection procedure appears to exceed the the two alternative methods, and our approach has ability to work effectively in real world applications.

Based on these change point estimates, the developmental data of *Drosophila Melanogaster* have divided into five segments. For each segment, we use the well-studied LASSO model in statistics to infer the causal relationship [21]. Specifically, let 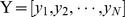, and 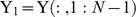, 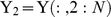, then reconstructing a static GRN can be formulized as follows,
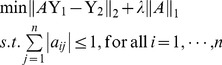
(16)The elements on each row of matrix 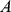 are independent and constraints in (16) are independent of each row in matrix 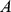. Therefore, optimization problem (16) can be reduced to 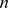 optimization problems, each having 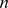 variables which are elements on a row of matrix 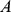. That is, for each row in matrix 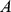, i.e., for each gene 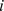 (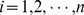), we have
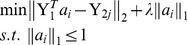
(17)in which, 
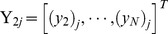
. By solving optimization problem (17), the topological structure of a GRN can be recovered.

**Figure 1:**
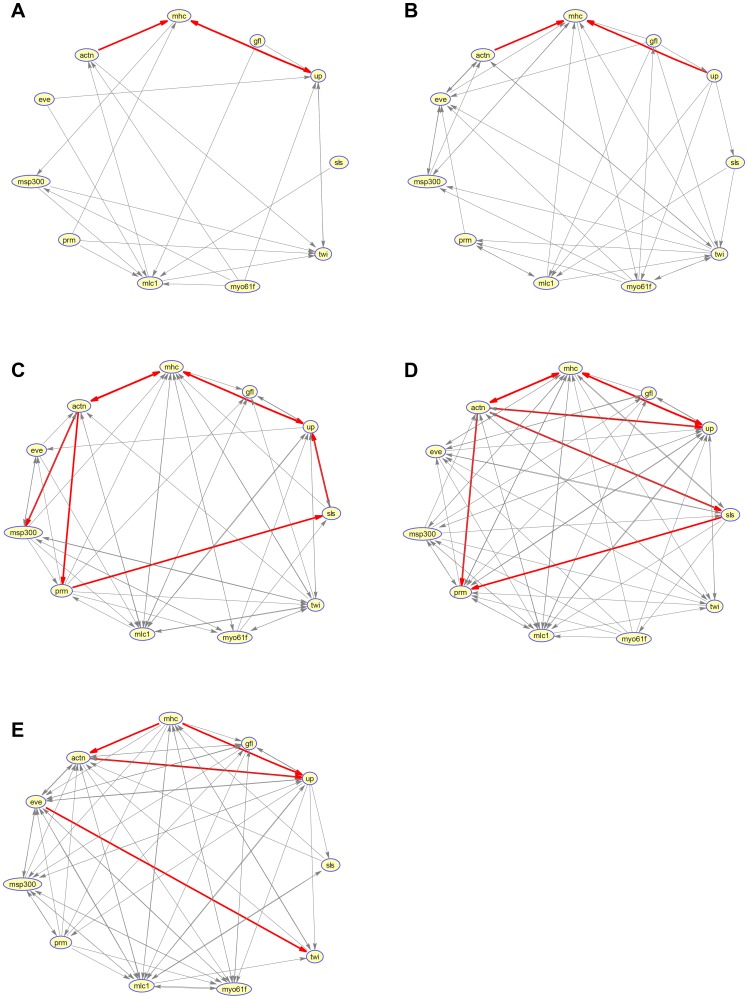
Gene regulatory networks recovered from gene expression time series of *Drosophila Melanogaster*. For each physiological stage, a network has been reconstructed(A: Adult, B: Embryo1, C: Embryo2, D: Larva, E: Pupa). And, interactions that have been verified are marked in red.

By setting 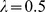, the topological structure for each segment is shown in Figure 1. An objective assessment of the reconstruction accuracy is not feasible due to the limited existing biological knowledge and the absence of a gold standard. However, we can mark some interactions that have been verified in red. More specifically, the interactions ‘actn
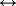
mhc’, ‘actn
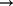
up’, and ‘up
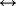
mhc’ have been verified in [26], [27]; the interaction ‘eve
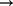
twi’ has been verified in [28]; and the interactions ‘actn
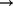
msp300’, ‘actn
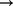
prm’, ‘prm
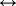
sls’ and ‘sls
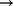
up’ have been verified in [29]; and the interaction ‘actn
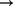
sls’ has been verified in [30]. These computation results using the developmental data of *Drosophila Melanogaster* show that the suggested two-step strategy may also be helpful in solving actual GRN reconstruction problem.

Apart from the developmental data of *Drosophila Melanogaster*, there exist some other real microarray compendia. Especially, the more recent DREAM5 network inference challenge offer some alternative real microarray compendia, which can be found in the web site at http://wiki.c2b2.columbia.edu/dream/index.php/D5c4 or in [31]. Whereas, the time series data in a single experiment are quite short. Thus, using them to reconstruct a simulative time-varying GRN that is obtained by concatenating gene expression data of different networks is very tricky. However, there exists an especial long time series, i.e., time series data of No. 49 experiment, Network4. This long time series has 48 samples; and from Sample 1 to Sample 11 there is no external interference to the network, while from Sample 12 to Sample 48 there is an uninterrupted external interference (P19) to the network. As mentioned before, biological networks change in response to environmental cues, and the change point is not always known *a priori* in general. Therefore, we use the suggested change point detecting algorithm to check whether there is a topological change in response to P19.

Based on the gold standard of Network4 suggested by the Dream project organizers, we select three sub-networks. In this way, although it is not clear that whether there is a biological significance for these sub-networks, it can be guaranteed that the system matrix 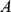 for each sub-network is not a zero matrix. The first one include 7 genes, which are G20, G61, G76, G111, G224, G273, G319. The second one include 8 genes, which are G15, G21, G45, G95, G101, G111, G212, G213. And, the third one include 8 genes, which are G15, G45, G47, G87, G101, G112, G152, G273. By setting 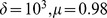 in Equations (11)–(15), we can obtain 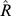 for the dataset. Then, setting 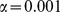 and using the change point detection procedure in Table 1, we find that each sub-network changes its network topology at the time interval 16 to 17. This result also verifies the general conclusion that biological networks are dynamic in response to environmental changes 6,7, and the rewiring processes may be time-delayed [11].

Finally, some information about the dataset can be found in [31]. More specifically, Network 4 is *S. cerevisiae*; the external interference P19 is phenelzine treatment; and the de-anonymized gene names are listed as follows: G15: YKL043W, G20: YJR147W, G21: YER045C, G45: YMR016C, G47: YNL167C, G61: YLR131C, G76: YJR060W, G87: YHR206W, G95: YNL314W, G101: YGL162W, G111: YOR028C, G112: YER111C, G152: YLR182W, G212: YDL106C, G213: YIL130W, G224: YEL009C, G273: YDR259C, and G319: YPR104C.

### Concluding Remarks

In this paper, we consider the time-varying GRN identification problem. The switching auto-regressive model is used to approximate the regulatory model for time-varying GRNs. And, a two-step strategy is proposed to recover the topological structure. In the first step, on the basis of a relation between the Kalman filter and recursive least squares estimation, it is shown that the innovation process is white if and only if the time series expression data are generated by the same sub-regulatory network. Based on this observation, a procedure is developed to detect change points of a time-varying GRN. The observed time series are divided into several segments based on these detection results; and each time series segment belonging to two successive demarcating change points is associated with an individual static regulatory network. Therefore, in the second step, for each time interval, the causal relationships inference problem can resort to conditional network structure identification methods, such as IOTA, and LASSO, etc.

The main difficulty of the time-varying GRN identification problem is that the identification problem includes a classification problem in which each data must be associated to the most suitable sub-network. The more precise the data classification is, the better the identification results are. The proposed two-step strategy efficiently estimates the change point, which results in the decoupling of the change point detection problem and the topology inference problem. Hence, the problem of identifying a time-varying regulatory model is transformed into that of identifying multiple single static regulatory models, which means that the proposed two-step strategy can ease the difficulty level in recovering a time-varying GRN. Simulation results show that the proposed strategy can detect the change point precisely and recover each individual topology structure effectively. Moreover, computation results with the developmental data of *Drosophila Melanogaster* show that the suggested strategy may also be helpful in solving actual GRN reconstruction problem.

Under our two-step strategy architecture, recovering a static GRN from time series is the most basic problem. However, this problem is not solved completely and efficaciously! Therefore, the most urgent problem is how to utilize gene expression time series data to obtain a static network structure with high accuracy.

## Supporting Information

Text S1 **Appendix: Proof of Theorem 1.**
(PDF)
