# Supplementary material for: A Kalman-Filter Based Approach to Identification of Time-Varying Gene Regulatory Networks
Source: PLoS One. 2013 Oct 7;8(10):e74571. doi: 10.1371/journal.pone.0074571 (PMC3792119; doi:10.1371/journal.pone.0074571)
Supplement: Text S1 — Appendix: Proof of Theorem 1. (PDF) [file pone.0074571.s001.pdf]

Appendix: Proof of Theorem 1

First, we demonstrate the conclusion that when $y_t|_{t=1}^N$ is generated by Equation (3), $e_t|_{t=1}^N$ is an independent random sequence.

To prove above conclusion, it is sufficient to show that $\mathbf{E}\left\{(e_i|Y_{i-1})(e_j|Y_{j-1})^T\right\} = \tilde{R}_i\delta_{ij}$ in which $\tilde{R}_i$ is a positive definite matrix. For this purpose, the following equation is at first established,

$$\mathbf{E}\left\{(e_i\,|Y_{i-1})\,(e_j\,|Y_{j-1})^T\right\} = 0, \qquad \forall i > j \tag{A.1}$$

Substitute the definition of e_t into this equation, the following relation can be directly obtained,

$$\begin{aligned}\mathbf{E}\left\{(e_i\,|Y_{i-1})\,(e_j\,|Y_{j-1})^T\right\} &= h_i\mathbf{E}\left\{\left(\left[x_{i-1}-\hat{x}_{i-1|i-1}\right]|Y_{i-1}\right)\left(\left[x_{j-1}-\hat{x}_{j-1|j-1}\right]|Y_{j-1}\right)^T\right\}h_j^T \\ &\quad +\mathbf{E}\left\{(w_i|Y_{i-1})(w_j\,|Y_{j-1})^T\right\} + h_i\mathbf{E}\left\{\left(\left[x_{i-1}-\hat{x}_{i-1|i-1}\right]|Y_{i-1}\right)(w_j\,|Y_{j-1})^T\right\} \\ &\quad + \mathbf{E}\left\{(w_i\,|Y_{i-1})\left(\left[x_{j-1}-\hat{x}_{j-1|j-1}\right]|Y_{j-1}\right)^T\right\}h_j^T\end{aligned} \tag{A.2}$$

Note that $\mathbf{E}\left\{(w_i|Y_{i-1})(w_j\,|Y_{j-1})^T\right\} = \mathbf{E}\{w_i\}\,\mathbf{E}^T\{w_j\} = 0$ for arbitrary $i \neq j$. Moreover,

$$\begin{aligned}\mathbf{E}\left\{(w_i\,|Y_{i-1})\left(\left[x_{j-1}-\hat{x}_{j-1|j-1}\right]|Y_{j-1}\right)^T\right\} &= \mathbf{E}\left\{w_i\left(\left[x_{j-1}-\hat{x}_{j-1|j-1}\right]|Y_{j-1}\right)^T\right\} \\ &= \mathbf{E}\{w_i\}\,\mathbf{E}^T\left\{\left[x_{j-1}-\hat{x}_{j-1|j-1}\right]|Y_{j-1}\right\} \\ &= 0\end{aligned} \tag{A.3}$$

Recall that in the updating procedure of Equations (5)-(7), K_{i-1} is completely determined when Y_{i-2} is given. We therefore have that

$$\mathbf{E}\left\{(K_{i-1}w_{i-1}\,|Y_{i-2})\left(\left[x_{j-1}-\hat{x}_{j-1|j-1}\right]|Y_{j-1}\right)^T\right\} = K_{i-1}\mathbf{E}\left\{(w_{i-1}\,|Y_{i-2})\left(\left[x_{j-1}-\hat{x}_{j-1|j-1}\right]|Y_{j-1}\right)^T\right\} = 0 \tag{A.4}$$

Using arguments similar to the above equation repeatedly, it can be proved that

$$\begin{aligned}&\mathbf{E}\left\{\left(\left[x_{i-1}-\hat{x}_{i-1|i-1}\right]|Y_{i-1}\right)\left(\left[x_{j-1}-\hat{x}_{j-1|j-1}\right]|Y_{j-1}\right)^T\right\} \\ =\;& \mathbf{E}\left\{\left(\left[(I-K_{i-1}h_{i-1})(x_{i-2}-\hat{x}_{i-2|i-2})-K_{i-1}w_{i-1}\right]|Y_{i-2}\right)\left(\left[x_{j-1}-\hat{x}_{j-1|j-1}\right]|Y_{j-1}\right)^T\right\} \\ =\;& (I-K_{i-1}h_{i-1})\mathbf{E}\left\{\left(\left[(x_{i-2}-\hat{x}_{i-2|i-2})\right]|Y_{i-2}\right)\left(\left[x_{j-1}-\hat{x}_{j-1|j-1}\right]|Y_{j-1}\right)^T\right\} \\ =\;& \cdots \\ =\;& (I-K_{i-1}h_{i-1})\cdots(I-K_jh_j)\mathbf{E}\left\{\left(\left[(x_{j-1}-\hat{x}_{j-1|j-1})\right]|Y_{j-1}\right)\left(\left[x_{j-1}-\hat{x}_{j-1|j-1}\right]|Y_{j-1}\right)^T\right\} \\ =\;& (I-K_{i-1}h_{i-1})\cdots(I-K_jh_j)\mathbf{E}\left\{\left[(x_{j-1}-\hat{x}_{j-1|j-1})\right]\left[x_{j-1}-\hat{x}_{j-1|j-1}\right]^T|Y_{j-1}\right\}\end{aligned} \tag{A.5}$$

Similarly, the following equation can be obtained

$$\mathbf{E}\left\{\left(\left[x_{i-1}-\hat{x}_{i-1|i-1}\right]|Y_{i-1}\right)(w_j\,|Y_{j-1})^T\right\} = -(I-K_{i-1}h_{i-1})(I-K_{i-2}h_{i-2})\cdots(I-K_{j+1}h_{j+1})K_jR \tag{A.6}$$

Substituting Equations (A.3), (A.5) and (A.6) into Equation (A.2), we have that

$$\mathbf{E}\left\{(e_i\,|Y_{i-1})\,(e_j\,|Y_{j-1})^T\right\} = h_i(I-K_{i-1}h_{i-1})(I-K_{i-2}h_{i-2})\cdots(I-K_{j+1}h_{j+1})\times \left\{(I-K_jh_j)\mathbf{E}\left\{(x_{j-1}-\hat{x}_{j-1|j-1})(x_{j-1}-\hat{x}_{j-1|j-1})^T\,|Y_{j-1}\right\}h_j^T-K_jR\right\} \quad (A.7)$$

On the other hand, from the properties of Kalman filtering, the next relation can be immediately established by Lemma 1.

$$\mathbf{E}\left\{(x_{j-1}-\hat{x}_{j-1|j-1})(x_{j-1}-\hat{x}_{j-1|j-1})^T\,|Y_{j-1}\right\} = P_{j-1|j-1} \quad (A.8)$$

Moreover, from the recursive procedure of Equations (5)-(7), it can be straightforwardly shown that $P_{j-1|j-1}h_j^T = K_jR + K_jh_jP_{j-1|j-1}h_j^T$. We therefore have that

$$(I-K_jh_j)\mathbf{E}\left\{(x_{j-1}-\hat{x}_{j-|j-1})(x_{j-1}-\hat{x}_{j-1|j-1})^T\,|Y_{j-1}\right\}h_j^T - K_jR = 0 \quad (A.9)$$

Equation (A.1) can now be obtained through combining Equations (A.7) and (A.9) together. Similarly, the following relation can also be proved

$$\mathbf{E}\left\{(e_i|Y_{i-1})(e_j|Y_{j-1})^T\right\} = 0,\ \forall i<j \quad (A.10)$$

Moreover, once again from properties of Kalman filtering, direct algebraic manipulations show that

$$\mathbf{E}\left\{e_ie_i^T\,|Y_{i-1}\right\} = R + h_iP_{i-1|i-1}h_i^T \quad (A.11)$$

Recall that R is assumed to be positive definite. From the above 2 equations and Equation (A.1), as well as the property that $P_{i-1|i-1}$ is positive semi-definite, it is apparent that $\tilde{R}_i = R + h_iP_{i-1|i-1}h_i^T$ satisfies the requirement. Based on this result and the relation $\mathbf{E}\{e_ie_j^T\} = \mathbf{E}\left\{\mathbf{E}\left\{(e_i|Y_{i-1})(e_j|Y_{j-1})^T\right\}\right\}$, it can be further proved that there exists a positive definite matrix $\bar{R}_i$, such that for arbitrary $i,j = 1,2,\cdots,N$,

$$\mathbf{E}\left\{e_ie_j^T\right\} = \bar{R}_i\delta_{ij} \quad (A.12)$$

Equation (A.12) means that when $y_t|_{t=1}^N$ is generated by Equation (3), $e_t|_{t=1}^N$ is an independent random sequence. To complete the other half of Theorem 1, we may consider the following time-varying GRN consisting of only two sub-networks,

$$y_t = A_{\lambda(t)}y_{t-1} + w_t, \qquad \lambda(t) = \begin{cases} 1, & t<t_s \\ 2, & t\geq t_s \end{cases} \quad (A.13)$$

From the above discussion, we know if $t_s > N$ or $t_s = 1$, it can be declared that the innovation process defined by Equation (8) is white. Assume now that $1 < t_s \leq N$. Define x_t as

$$x_t = \begin{cases} \mathbf{vec}\left(A_1^T\right), & 1\leq t<t_s \\ \mathbf{vec}\left(A_2^T\right), & t_s\leq t\leq N \end{cases} \quad (A.14)$$

Then, y_t of Equation (A.13) can still be expressed as $y_t = h_tx_t + w_t$, in which h_t has the same definition as that of Equation (4). Consider the correlation matrix between random vectors e_{t_s} and e_j with $1\leq j<t_s$. From the definition of e_t, we have that

$$\begin{aligned}\mathbf{E}\left[e_{t_s}e_j^T\right] &= \mathbf{E}\left\{[h_{t_s}x_{t_s} - h_{t_s}\hat{x}_{t_s-1|t_s-1} + w_{t_s}][h_jx_j - h_j\hat{x}_{j-1|j-1} + w_j]^T\right\} \\ &= \mathbf{E}\left\{[h_{t_s}x_{t_s-1} - h_{t_s}\hat{x}_{t_s-1|t_s-1} + w_{t_s}][h_jx_j - h_j\hat{x}_{j-1|j-1} + w_j]^T\right\} + \mathbf{E}\left\{h_{t_s}(x_{t_s}-x_{t_s-1})e_j^T\right\}\end{aligned} \quad (A.15)$$

Note that x_i remains unchanged whenever $1 \leq i \leq t_s - 1$. It can therefore be declared from above discussion that

$$\mathbf{E}\left\{[h_{t_s}x_{t_s-1} - h_{t_s}\hat{x}_{t_s-1|t_s-1} + w_{t_s}][h_j x_j - h_j\hat{x}_{j-1|j-1} + w_j]^T\right\} = 0 \tag{A.16}$$

From the definitions of h_t and x_t, it can be directly proved that $h_{t_s}(x_{t_s} - x_{t_s-1}) = (A_2 - A_1)y_{t_s-1}$. Direct algebraic operations show that

$$\mathbf{E}[h_{t_s}(x_{t_s} - x_{t_s-1})e_j^T] = (A_2 - A_1)\mathbf{E}\left(y_{t_s-1}e_j^T\right) \tag{A.17}$$

Note that for arbitrary $j = 1, 2, \cdots, N$,

$$\begin{aligned}\mathbf{E}(e_j) &= \mathbf{E}\left\{\mathbf{E}(e_j|Y_{j-1})\right\} \\ &= \mathbf{E}\left\{\mathbf{E}[h_j(x_j - \hat{x}_{j-1|j-1}) + w_j|Y_{j-1}]\right\} \\ &= \mathbf{E}\left\{h_j\mathbf{E}[(x_j - \hat{x}_{j-1|j-1})|Y_{j-1}]\right\} + \mathbf{E}\left\{\mathbf{E}(w_j|Y_{j-1})\right\} \\ &= 0\end{aligned} \tag{A.18}$$

Moreover, if $j \geq t_s - 1$, then,

$$\begin{aligned}\mathbf{E}\left(y_{t_s-1}e_j^T\right) &= \mathbf{E}\left\{\mathbf{E}\left(y_{t_s-1}e_j^T|Y_{t_s-1-1}\right)\right\} \\ &= \mathbf{E}\left\{\mathbf{E}\left[(h_{t_s-1}\hat{x}_{t_s-1-1|t_s-1-1} + e_{t_s-1})e_j^T|Y_{t_s-1-1}\right]\right\} \\ &= \mathbf{E}\left\{h_{t_s-1}\hat{x}_{t_s-1-1|t_s-1-1}\mathbf{E}^T\left[e_j|Y_{t_s-1-1}\right]\right\} + \mathbf{E}\left\{\mathbf{E}\left(e_{t_s-1}e_j^T|Y_{t_s-1-1}\right)\right\} \\ &= \mathbf{E}\left\{e_{t_s-1}e_j^T\right\} \\ &= \bar{R}_{t_s-1}\delta_{t_s-1,j}\end{aligned} \tag{A.19}$$

in which $\bar{R}_i$ has the same definition as that of Equation(A.12). Therefore, if $t_s > j \geq t_s - 1$, then, we have that

$$\mathbf{E}[h_{t_s}(x_{t_s} - x_{t_s-1})e_j^T] = (A_2 - A_1)\bar{R}_{t_s-1}\delta_{t_s-1,j} \tag{A.20}$$

As $\bar{R}_{t_s-1}$ is proved to be positive above, it can therefore be declared that if $A_2 \neq A_1$, then, it is certain that there is at least one $1 \leq j \leq N$ satisfying $j \neq t_s$ and

$$\mathbf{E}[h_{t_s}(x_{t_s} - x_{t_s-1})e_j^T] \neq 0 \tag{A.21}$$

Substituting Equations (A.16) and (A.21) into Equation (A.15), we have that for the aforementioned j, $\mathbf{E}(e_{t_s}e_j^T) \neq 0$, which means that gene expression time series data are generated by multiple sub-networks, the innovation process $e_t|_{t=1}^N$ defined by Equation (8) is no longer white. This completes the proof. $\Diamond$
